# Editorial: Insights in thermophilic microbes: from OMICS to bioactive compounds

**DOI:** 10.3389/fmicb.2023.1206185

**Published:** 2023-05-16

**Authors:** Aparna Banerjee, Laura Zucconi, Rajesh K. Sani

**Affiliations:** ^1^Instituto de Ciencias Químicas Aplicadas, Facultad de Ingeniería, Universidad Autónoma de Chile, Talca, Chile; ^2^Department of Ecological and Biological Sciences, University of Tuscia, Viterbo, Italy; ^3^Department of Chemical and Biological Engineering, South Dakota School of Mines and Technology, Rapid City, SD, United States

**Keywords:** thermophile, OMICS, bioactive compounds, biotechnological application, genome

Exploring the research frontiers in extremophilic microbiology is critical in this time of global warming and ever-increasing climate change impacts, considering the risk of species loss in vulnerable or rare ecosystems. Studying the extremophilic microbiotechnology is also important for possible industrial applications (Banerjee et al., 2020). Microbial life adapts to environmental stresses in extreme environments, such as extreme salinity, pH, temperature and pressure, radiation, and toxic compounds. While temperature is a major limiting factor for microbial life, thermophiles live in high-temperature ecosystems all over the world. Different types of thermophilic bacteria, archaea, fungi, and algae thrive in high-temperature habitats, such as hot springs, hydrothermal vents, geysers, fumaroles, or mud volcanos (Merino et al., 2019). Understanding life in high-temperature environments requires exploring microbial diversity, interactions, functioning, and adaptations to environmental extremes. To accommodate high-temperature stress, thermophiles are modifying the cellular microenvironment by producing thermostable enzymes and bioactive compounds, for example, polysaccharides, polyesters, and pigments (Arena et al., 2009). The study of thermophiles and the molecular strategies they have developed to tackle extreme temperatures may also provide new insights into the production of some biotechnologically important metabolites (Shivlata and Satyanarayana, 2015). It is time to explore these capabilities using cutting-edge molecular biology, analytical chemistry, and bioinformatics methods. Finally, thermophiles include some of the most deeply branched lineages of life on Earth, and understanding their biology provides context for imprints of life from billions of years ago, and for environmental limits for life on Earth and exoplanets. In this Research Topic, thermophilic bacteria, cyanobacteria, and heat-tolerant marine microbial communities have been investigated focusing on genome-, secretome-, and transcriptome-related insights, to better understand heat and metal tolerances of thermophiles, and to explore their biotechnological potential.

Bhalla et al. have discussed the prospect of thermophilic microbiotechnology via *Geobacillus* sp. strain WSUCF1 containing different carbohydrate-active enzymes (CAZymes) that specifically hydrolyze hemicellulose in lignocellulosic biomass. They have used proteomic, genomic, and bioinformatic tools to analyze the genome regarding the relative abundance of cellulolytic, hemicellulolytic, and lignin-modifying enzymes present in its secretome. The CAZyme profiles of secretomes differed based on substrates and complexity, composition, and pretreatment conditions. The enzyme activity of secretomes also altered depending on the substrate used. Based on these data, the secretomes were used with commercial and purified enzymes to achieve the saccharification of ammonia fiber expansion (AFEX)-pretreated corn stover and extractive ammonia (EA)-pretreated corn stover. This work opens the opportunity of generating thermophilic CAZymes in a biorefinery using economical substrates, such as AFEX-pretreated corn stover, reducing the use of costly enzyme processing steps that are used in enzyme production.

Mukherjee et al. have given insight into the genome of a thermophilic cyanobacterium. They focused on the stress response of a filamentous, AT-rich, heterocystous cyanobacterium, *Mastigocladus laminosus* UU774, isolated from an Indian hot spring. The genome contained an essential scaffold_38 fragment of unknown origin associated with severe nitrogen and nutrition stress. Persistent exposure to nitrogen compounds during starvation exhibited adverse effects, leading to loss of mobility, less ability to fight pathogens, reduced cell division, decreased nitrogen-fixing ability, reduced capacity to form biofilms, decreased photosynthetic and light-sensing ability, and reduced production of secreted effectors and chromosomal toxin genes. During the presence of nitrogen, reduced expression of key enzymes involved in the uptake of phosphate and enzymes protecting oxygen-sensitive nitrogenases was observed. UU774 endures heat by overexpressing peptidases that may be degrading abnormally folded proteins produced during heat. The work suggested *M*. *laminosus* UU774 has diverged from *Fischerella* sp. PCC 9339, another hot spring species isolated in the United States.

Some articles in this Research Topic also covered the extremophilic adaptations of different thermophiles, regarding the coexistence of heavy metal tolerance and antibiotic resistance (Najar et al.), and heat response of endospores (Chakraborty et al.). Najar et al. explored diversity, antibiotic and heavy metal resistance, and the prevalence and presence of antibiotic resistance gene (ARG) and metal resistance gene (MRG) in *Geobacillus* species. All thermophilic *Geobacillus* species were sensitive to at least 10 different antibiotics, as proven by the lack of ARGs in the whole genome. Some of these isolates were resistant to at least five different heavy metals, and whole genome sequencing revealed the presence of MRGs too. In the article by Chakraborty et al., community responses to sudden changes in temperature and pressure of different populations have been observed. In this context, incubation of permanently cold Arctic fjord sediments at 50°C for 216 h with and without volatile fatty acid amendment triggered major changes in community structure. The germination of thermophilic endospores from the sediment was traced. Comparing community similarity at different intervals of the incubations showed distinct temporal shifts in microbial populations, depending on organic substrate alteration. Metabolite patterns indicated that amino acids and other sediment-derived organics were decomposed by fermentative *Clostridia* within the first 12–48 h of incubation. The resulting exponential increases in sulfate reduction highlight the importance of volatile fatty acids as electron donors for different sulfate-reducing *Desulfotomaculia* populations. The succession of germinated endospores activated by sudden exposure to high temperatures and controlled by nutrient availability can offer a future model for understanding the ecological response of dormant microbial communities under environmental alterations.

Some other works have given detailed insights into the genome of thermophilic *Geobacillus*, and *Parageobacillus* species focusing on their potential biotechnological and industrial applications. Yildiz et al. have reported the details of the *Parageobacillus thermantarcticus* strain M1 genome, isolated from the geothermal soil of the crater of Mount Melbourne. Strain M1 demonstrated biotechnological and industrial potential owing to its ability to produce exopolysaccharides (EPSs), ethanol, and thermostable extracellular enzymes, such as xylanase and β-xylosidase, and intracellular ones, such as xylose/glucose isomerase and protease. Thermophilic spore forming *Geobacillus* sp. CX412 isolated from hot spring soil in China was reported by Li et al.. COG, KEGG, and CAZymes analysis demonstrated that *Geobacillus* sp. CX412 was a highly adaptive strain with a high number of 73 annotated transposons in the genome, which is relatively rare in *Geobacillus*. Compared to the near-derived strains of *Geobacillus* and *Parageobacillus*, it was found that *Geobacillus* sp. CX412 has unique β-lactam resistance and more active metabolism. Additionally, its genome encodes glycoside hydrolases and other genes related to lignocellulose breakdown, suggesting that *Geobacillus* sp. CX412 has a considerable biomass degradation potential.

Altogether, the original research articles published in this Research Topic provide new insights into the physiology of thermophilic microorganisms, their environmental adaptations and tolerances, genome-to-secretome linkages, and biotechnological or industrial potential.

## Author contributions

All authors listed have made a substantial, direct, and intellectual contribution to the work and approved it for publication.
